# Early Onset Obesity and Risk of Metabolic Syndrome Among Chilean Adolescents

**DOI:** 10.5888/pcd14.170132

**Published:** 2017-10-12

**Authors:** Lorena Sonia Pacheco, Estela Blanco, Raquel Burrows, Marcela Reyes, Betsy Lozoff, Sheila Gahagan

**Affiliations:** 1 University of California San Diego–San Diego State University Joint Doctoral Program in Public Health, La Jolla, California; 2 Division of Child Development and Community Health, Department of Pediatrics, University of California, San Diego, California; 3 University of Chile Doctoral Program in Public Health, Santiago, Chile; 4 Public Health Nutrition Unit, Institute of Nutrition and Food Technology (INTA), University of Chile, Santiago, Chile; 5 Center for Human Growth and Development, University of Michigan, Ann Arbor, Michigan; 6 Department of Pediatrics and Communicable Diseases, University of Michigan, Ann Arbor, Michigan

## Abstract

**Introduction:**

Obesity and metabolic syndrome (MetS) indicators have increased globally among the pediatric population. MetS indicators in the young elevate their risk of cardiovascular disease and metabolic disorders later in life. This study examined early onset obesity as a risk factor for MetS risk in adolescence.

**Methods:**

A cohort of Chilean participants (N = 673) followed from infancy was assessed at age 5 years and in adolescence (mean age, 16.8 y). Adiposity was measured at both time points; blood pressure and fasting blood samples were assessed in adolescence only. Early onset obesity was defined as a World Health Organization *z* score of 2 standard deviations (SDs) or more for body mass index (BMI) at age 5 years. We used linear regression to examine the association between early onset obesity and adolescent MetS risk *z* score, adjusting for covariates.

**Results:**

Eighteen percent of participants had early onset obesity, and 50% of these remained obese in adolescence. Mean MetS risk *z* score in adolescence was significantly higher among those with early onset obesity than among those without (1.0; SD, 0.8 vs 0.2; SD, 0.8 [*P* < .001]). In the multivariable model, early onset obesity independently contributed to a higher MetS risk score in adolescence (β = 0.27, *P* < .001), controlling for obesity status at adolescence and sex, and explained 39% of the variance in MetS risk.

**Conclusion:**

Early onset obesity as young as age 5 years relates to higher MetS risk.

**Editor’s Note:** This article is 1 of 2 winners of *PCD*‘s 2017 Student Research Paper Contest in the Doctoral category.

## Introduction

Obesity among children is a global public health problem (1,2), and signs of metabolic syndrome (MetS) have increased among both children and adolescents over the past 25 years (3,4). MetS is defined as having at least 3 of 5 risk factors: a large waist circumference, high blood pressure, fasting hyperglycemia, hypertriglyceridemia, and low high-density lipoprotein (HDL) cholesterol levels (5). Although few children meet all 5 MetS criteria, up to 30% of obese children have at least one element of MetS (6). A recent systematic review showed a median prevalence of MetS of 3% (range 0%–19.2%) among all children and 29% (range 10%–66%) among obese children (3). In a sample of US adolescents aged 12 to 17 years, the overall MetS prevalence was 7%, with a range from 19% to 35% among obese adolescents (7). Additionally, Hispanic adolescents had a higher MetS prevalence (11%) than non-Hispanic white adolescents (9%) (8).

MetS increases a person’s risk for developing chronic disease (9,10). Pediatric MetS is independently associated with type 2 diabetes and adult MetS and with subclinical atherosclerosis leading to cardiovascular disease (CVD) (11,12). Additionally, research shows that obesity tracks from childhood into adulthood (13) and contributes to adverse consequences, including premature mortality and cardiometabolic disorders (14,15). However, the impact of the age of obesity onset in early childhood on adolescent MetS risk has not been documented, because research has largely focused on infant weight gain and catch-up growth as predictors of health outcomes later in life (16–18). This study examined early onset obesity as a risk factor for MetS risk in adolescence. We hypothesized that obesity onset early in life is associated with a higher MetS risk score in adolescence.

## Methods

### Study design and population

Participants were 677 Chilean infants who were part of an observational longitudinal study of biopsychosocial determinants of obesity and CVD risk. From 1991 through 1996, 1,933 infants were enrolled in either a preventive trial of iron supplementation to prevent iron-deficiency anemia or a neuromaturation study, a study that assessed neurodevelopment by using neurophysiological and electrophysiological techniques. The studies were conducted in Santiago, Chile, where infancy iron deficiency was widespread at the time and no national program existed for iron supplementation. Infants were from low-to-middle income, working-class communities. Inclusion criteria for the infancy studies were an uncomplicated, singleton, term, vaginal birth with birthweights of 3 kg or more, no major congenital abnormalities, and no prior iron therapy. Because of a successful national breastfeeding campaign, all but 8 infants in the cohort were initially breastfed.

Infants were recruited at age 4 to 6 months. Infants without iron-deficiency anemia were randomly assigned to high-iron supplementation, low-iron supplementation, or usual nutrition (no added iron). Further details about enrollment and trial specifications are described elsewhere (19). A total of 1,657 infants completed the preventive trial (high iron, n = 718; low iron, n = 405; usual nutrition, n = 534). Infants found to have iron-deficiency anemia, and the next nonanemic infant (the control), were treated with medicinal iron and participated the neuromaturation study (20). A total of 135 infants completed the neuromaturation study. At age 5 years, because of a cut in funding, only 2 of the 3 randomly selected preventive trial groups and neuromaturation trial participants could be evaluated. Thus, only 888 of 1,501 infants who were in the high-iron and no-added-iron groups or completed the neuromaturation study were assessed. At age 16 years, participants from the 5-year follow up were invited to participate in a study of obesity and CVD risk. A total of 677 of 888 (76%) participants agreed and were assessed from 2009 through 2012. Our analytic sample for the obesity and MetS study consisted of 673 participants from the obesity and CVD risk study who had complete data at 5 years and 16 years (Figure).

**Figure:**
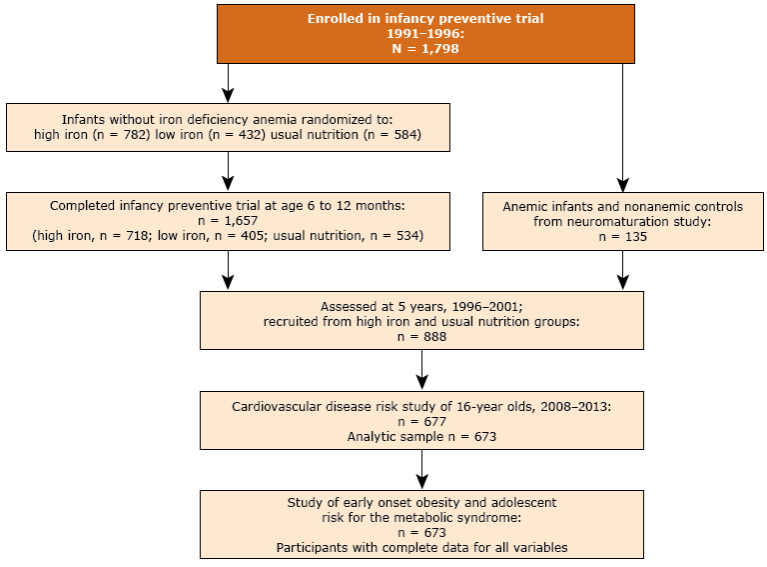
Flow of participants in study on relationship between early onset obesity and metabolic syndrome risk in adolescence, Santiago, Chile, 2009–2012. Participants were drawn from a larger study of infancy iron-deficiency anemia.

The sample was representative of the original cohort, with no differences in infant and family characteristics, including birthweight (3.5 kg in the original cohort vs 3.6 kg in the final analytic sample), breastfeeding for at least 6 months (63% in the original cohort vs 61% in the final analytic sample), socioeconomic status (SES) (27.3 on the Graffar index [21] for the original cohort vs 27.0 for the final analytic sample), and household environment (30.1 on the Home Observation for Measurement of the Environment [HOME] [22] scale for the original cohort vs 30.0 for the final analytic sample). The study was approved by the institutional review boards of the University of California, San Diego, the University of Michigan, and the University of Chile Institute of Nutrition and Food Technology (INTA).

### Data collection and analysis

Participants were considered to have early onset obesity if obesity was present at age 5 years (defined as ≥2 standard deviations [SDs] for body mass index [BMI] *z* score) by using WHO growth standard indicators. BMI is a measure of weight relative to height (kg/m^2^), with age-specific and sex-specific norms (23).

Adolescents were assessed at age 16 to 17 years. Height (cm), weight (kg), waist and hip circumference (cm), and blood pressure (mmHg) were measured by a physician-investigator at INTA. Standardized procedures were used to measure weight to the closest 0.1 kg by using a SECA scale (SECA), and height to the closest 0.1 cm by using a Holtain stadiometer (Holtain Ltd) (24). Each measurement was taken twice, and a third measurement was taken if the difference between the first 2 exceeded 0.3 kg for weight, 0.5 cm for height, or 1.0 cm for waist. The WHO BMI *z* score indicator was used to dichotomize (yes/no) obesity status at adolescence, with obesity defined as an SD of 2 or more in the BMI *z* score. Fasting serum triglyceride, cholesterol, and glucose levels were assessed. Serum glucose concentration (mg/dL) was determined by using an enzymatic–colorimetric test (Química Clínica Aplicada S.A.). Triglyceride (mg/dL), and cholesterol (mg/dL) levels were determined with the Vitros dry analytical methodology (Ortho Clinical Diagnostics Johnson & Johnson Inc).

A continuous MetS risk *z* score was calculated by applying the equations developed by Gurka et al (25). The equations provide a sex-specific and race-specific *z* score measure for MetS risk based on standardized and log-transformed values for each component of the MetS.

Characteristics that may be associated with both the variable of interest and the outcome were considered covariates. For infancy, the following were considered: birthweight, SES, breastfeeding, emotional and material support provided in the home environment, iron status during infancy, and iron supplementation as part of the preventive trial. For adolescents, the following were potential covariates: age at menarche, age at the adolescent assessment, physical activity, and obesity status. Birthweight, measured in kilograms, was analyzed as a continuous measure. Iron status during infancy, coded as iron sufficient, iron deficient, or iron-deficient anemic, was dichotomized to iron sufficient (0) and iron deficient or iron-deficient anemic (1) for modeling purposes. Iron supplementation in infancy was dichotomized to iron supplementation (high or low) (1) and no iron supplementation (0). The SES variable, the Graffar index, is a pseudocontinuous variable based on a 13-item questionnaire that produces a composite score that comprises questions on mothers’ and fathers’ years of education, occupation, and income (21) the higher the Graffar index, the lower the SES. Questions were coded as absent (1) to plentiful (6), for a possible score range of 13 to 78. The quality of the home environment that supported the children’s development was assessed with HOME, a 45-item, observer-rated checklist (22). A higher HOME score reflects a more supportive home environment for children’s development. Scores range from 0 to 45. Physical activity at adolescence was measured by using a 5-item questionnaire, validated for use in young populations (26). The questionnaire addresses planned and unplanned physical and sedentary activities as a continuous score between 0 and 10. Age at menarche and age at adolescent measurement were analyzed as continuous variables.

### Statistical analyses

SAS version 9.2 (SAS Institute) was used for all statistical analyses, with the exception of computed MetS risk score, for which SPSS version 22.0 (IBM Corp) was used.

For describing sample characteristics, continuous variables were expressed as mean and SD, and categorical variables were expressed as frequencies. All variables were assessed for normality. Unadjusted comparisons between early onset obesity groups were calculated by using *t* tests for continuous outcomes and χ^2^ tests for categorical outcomes. Regression diagnostics, using tests and graphical methods, examined linear regression assumptions including linearity (residual vs predictor plot) normality (Shapiro–Wilk test), homogeneity of variance (Breusch–Pagan test) and independence (Durbin–Watson statistic). All of these assumptions were met, indicating linearity, uncorrelated and normally distributed estimated residuals with constant variance. Influence and collinearity were also examined, with no extreme deviations observed for studentized and jackknife residuals, and small leverage and Cook’s distance values. Multivariable linear regression analysis was used to assess the relationship between early onset obesity and MetS risk score, adjusting for possible confounders. The full model was tested by using backward elimination. Variables that were not statistically associated with the dependent variable were manually removed. Because the study sample participated in a preventive trial, iron status during infancy and iron supplementation were initially included as covariates. Neither variable was significantly associated with the outcome, and thus both were removed from the final model. Age at menarche, which was tested in the multivariable model, was not significantly associated with outcome and therefore was dropped from the final model. Significance was set at a *P* value of less than .05. Multicollinearity between variables was assessed with tolerance level with a cut point of less than 0.10. There was no evidence of multicollinearity in the model.

Marginal structural models (MSMs) refine the adjustment made by traditional analytic approaches and predict an estimate that accounts for the bias that exists when time-dependent covariates might act as both confounders and intermediates in a linear association. We used an MSM as a sensitivity analysis to account for potential bias resulting from the inclusion of adolescent weight status in the final model; bias may arise because adolescent weight status both mediates and confounds the relationship between early-life obesity and adolescent MetS risk score. To carry out these analyses, we estimated stabilized inverse probability weights (27) and reweighted our sample to create a pseudopopulation in which the exposure, early onset obesity, is statistically independent of potential time-dependent confounders. Results from the pseudopopulation models supported initial findings, indicating limited bias resulting from the inclusion of adolescent weight status as a covariate in the multivariable linear regression model.

## Results

Mean age of participants at adolescence was 16.8 years, and 52.9% were male (Table 1). The mean birthweight in the study population was 3.6 kg (SD, 0.4 kg). Early onset obesity was found in 18.1% of the participants, of which 41.0% were girls. We found no significant differences in birthweight, sex, SES, HOME scores, and physical activity in adolescence between participants with early onset obesity and participants without early onset obesity. Obesity status at adolescence was related to early onset obesity. Of those with early onset obesity, 50% were obese at adolescence, in contrast to 6% of the comparison group (*P* < .001).

**Table 1:** Characteristics of Participants (N = 673), Study of Relationship Between Early Onset Obesity and Risk of Metabolic Syndrome Among Adolescents^a^, Santiago, Chile, 1991–1996 and 2009–2012

Characteristic	Total Sample (n = 673)	Early Onset Obesity (n = 122)	No Early Onset Obesity (n = 551)	*P* Value^b^
**Infancy**				
Birthweight, mean (SD), kg	3.6 (0.4)	3.6 (0.4)	3.5 (0.4)	.67
Male, %	52.9	59.0	51.5	.13
Breastfeed ≥6 months, %	63.4	63.6	63.3	.96
**Iron status during infancy, %**				
Iron sufficient	41.5	34.4	43.0	.06
Iron deficient	40.1	40.2	40.1	
Iron deficiency anemia	18.4	25.4	16.9	
**Socioeconomic status, Graffar index^c^, mean (SD)**	27.0 (6.3)	27.0 (6.2)	27.0 (6.3)	.87
**HOME score^d^, mean (SD)**	30.2 (4.7)	30.2 (4.8)	30.2 (4.7)	.90
**Supplementation group, %**				
High iron	47.2	45.9	47.6	.96
Low iron	2.7	3.3	2.5	
No added iron	42.1	42.6	41.9	
Neuromaturation study^e^	8.0	8.2	8.0	
**Adolescence**				
Age at menarche^f^, mean (SD), y	12.5 (1.4)	12.0 (1.4)	12.5 (1.4)	.01
Age at adolescent measurement, y	16.8 (0.3)	16.8 (0.3)	16.8 (0.3)	.48
Physical activity score^g^, mean (SD)	4.1 (1.6)	4.0 (1.5)	4.1 (1.7)	.38
Obesity at 16 y^h^, %	14.1	50.0	6.2	<.001

Abbreviations: SD, standard deviation; HOME, home observation for measurement of the environment.

a Early onset obesity defined as obese at 5 years of age following World Health Organization *z* score cut-off ≥2 SD body mass index (kg/m^2^).

b *P* value for χ^2^ test for categorical variables and *t* test for continuous variables.

c Graffar index is a social stratification tool used to assess socioeconomic status (range 13-78); the higher the Graffar index, the lower the socioeconomic status (21).

d HOME score (range 0-45) is a home environment quality assessment tool; the higher the HOME score, the better the home environment for child development (22).

e Participants in the neuromaturation study were infants found to have iron-deficiency anemia at age 6 months, and the next nonanemic infant (control) whose neurodevelopment was evaluated with neurophysiological and electrophysiological techniques (20).

f Female participants only.

g Physical activity score (range 0-10) assessing planned and unplanned physical and sedentary activities (26).

h Obesity at 16 years defined as obese at 16-year follow-up according to the World Health Organization z score cut-off ≥2 SD for body mass index.

The MetS risk score and all variables related to CVD risk were significantly higher in the early onset obesity group, compared with the group without early onset obesity, with the exception of HDL cholesterol, which was inversely related to CVD risk, and fasting blood glucose, which did not differ between groups (Table 2). Participants in the early onset obesity group had significantly higher mean total cholesterol levels (156.4 mg/dL; SD, 27.9 vs 151.1 mg/dL; SD 27.5, *P* = .04) and low-density lipoprotein cholesterol levels (98.9 mg/dL, SD 24.8 vs 93.3 mg/dL, SD 24.2, *P* = .03) compared with the group without early onset obesity. Additional analyses for MetS components by sex showed that adolescent boys had significantly lower mean total cholesterol and HDL cholesterol levels and significantly higher fasting blood glucose levels and blood pressure than adolescent girls.

**Table 2:** Metabolic Syndrome Risk Score^a^ and Cardiovascular Disease Risk Factors Among Participants (N = 673), Study of Relationship Between Early Onset Obesity^b^ and Risk of Metabolic Syndrome Among Adolescents, Santiago, Chile 1991–1996 and 2009–2012^c^

Variable	Total Population (N = 673)	Early Onset Obesity (n = 122)	No Early Onset Obesity (n = 551)	*P* Value^d^
Metabolic syndrome risk *z *score	0.3 (0.8)	1.0 (0.8)	0.2 (0.8)	<.001
Waist circumference, cm	81.3 (11.4)	94.0 (12.5)	78.5 (9.0)	<.001
HDL cholesterol, mg/dL	40.2 (10.6)	37.1 (9.5)	40.9 (10.7)	<.001
Triglycerides, mg/dL	88.2 (50.0)	103.3 (57.2)	84.8 (47.7)	.001
Systolic blood pressure, mm Hg	111.7 (10.5)	117.5 (11.7)	110.4 (9.8)	<.001
Diastolic blood pressure, mm Hg	69.1 (7.1)	71.9 (7.2)	68.5 (6.9)	<.001
Fasting blood glucose, mg/dL	88.6 (9.5)	89.5 (12.2)	88.5 (8.8)	.36

Abbreviations: HDL, high-density lipoprotein.

a Metabolic syndrome risk *z *score calculated with sex-specific and race-specific equations with confirmatory factor analysis.

b Early onset obesity defined as obese at age 5 years according to World Health Organization z score cut-off ≥2 standard deviations for body mass index (kg/m^2^).

c Values are unadjusted mean (standard deviation) unless otherwise indicated.

d *P* values calculated with *t* test for continuous variables for differences between the early onset obesity group and no early onset obesity group.

The final model, controlling for sex and obesity status in adolescence, indicated that early onset obesity was associated with a higher MetS risk score in adolescence (β = 0.27; 95% confidence interval [CI] 0.13– 0.41, *P* < .001) (Table 3) and explained 39% of the variance in the MetS risk score. The adjusted mean and standard error (SE) MetS risk score was 1.0 (SE, 0.06) and 0.7 (SE, 0.04) for participants with and without early onset obesity, respectively. Additionally, female sex was associated with a lower MetS risk score in the model (β = −0.26; 95% CI, −0.35 to −0.17; *P* < .001), adjusting for other covariates.

**Table 3:** Linear Regression Models to Determine Adjusted Associations With Participants’ (N = 673) Metabolic Syndrome Risk Score^a^, Study of Relationship Between Early Onset Obesity and Risk of Metabolic Syndrome Among Adolescents, Santiago, Chile 2009–2012

Variable	Full Model^b^		Final Model^b^	
	β (95% CI)	*P* Value^c^	β (95% CI)	*P* Value^c^
Early onset obesity^d^	0.29 (0.15 to 0.44)	<.001	0.27 (0.13 to 0.41)	<.001
Obesity at 16 y^e^	1.18 (1.02 to 1.34)	<.001	1.20 (1.04 to 1.35)	<.001
Female^f^	−0.29 (−0.40 to −0.18)	<.001	−0.26 (−0.35 to −0.17)	<.001
Birthweight, kg	0.08 (−0.05 to 0.21)	.23	—	—
Breastfed ≥6 months	0.01 (−0.09 to 0.11)	.83	—	—
Iron deficient during infancy^g^	−0.02 (−0.12 to 0.08)	.73	—	—
Iron supplementation^h^	0.05 (−0.05 to 0.15)	.35	—	—
Graffar index^i^	0.004 (−0.01 to 0.01)	.32	—	—
HOME score^j^	0.003 (−0.01 to 0.01)	.54	—	—
Age at menarche^k^	−0.01 (−0.06 to 0.05)	.83	—	—
Age at adolescent measurement	−0.02 (−0.20 to 0.18)	.86	—	—
Physical activity score^l^	−0.03 (−0.06 to 0.01)	.10	—	—

Abbreviations: —, not calculated for parsimony; CI, confidence interval; HOME, home observation for measurement of the environment.

a Metabolic syndrome risk score calculated with sex-specific and race-specific equations with confirmatory factor analysis.

b Linear regression modeling, presenting β estimate and 95% confidence interval.

c *P* values calculated by linear regression modeling, adjusted for all other listed variables in each of the models (full and final).

d Early onset obesity defined as obese at age 5 years according to World Health Organization *z* score cut-off ≥2 standard deviations for body mass index (kg/m^2^). Reference: no early onset obesity.

e Obesity at age 16 years defined as obese at 16-year follow-up according to World Health Organization z score cut-off ≥2 standard deviations for body mass index. Reference: no obesity at age 16 years.

f Reference: male.

g Includes iron deficient and iron deficiency anemia. Reference: iron sufficient.

h Iron supplementation includes: high-iron and low-iron supplementation during trial. Reference: no iron supplementation.

i Graffar index is a social stratification tool used to assess socioeconomic status (range 13-78); the higher the Graffar index, the lower the socioeconomic status (21).

j HOME score (range 0-45) is a home environment quality assessment tool; the higher the HOME score, the better the home environment for child development (22).

k Age at menarche for female participants.

l Physical activity score (range 0-10) assessing planned and unplanned physical and sedentary activities (26).

Findings from the MSM, a sensitivity analysis, did not differ from findings of the multivariable regression analysis. This corroborated the effect size of early onset obesity and its relationship with MetS risk score in adolescence.

## Discussion

This study showed that early onset obesity was associated with greater MetS risk in adolescence. Independent of adolescent obesity status and sex, a child who had obesity at age 5 years had a higher MetS risk score (β = 0.27) at age 16. These results support our initial hypothesis.

Our findings are consistent with those in a mid-childhood cohort (28). Using a similar analytic approach and focusing on metabolic profiles that included dyslipidemia, hypertension, and insulin resistance, Garnett et al. (28) concluded that children who were overweight or obese at age 8 years were almost 7 times as likely to have CVD risk-clustering at age 15 years as those who were not overweight or obese (odds ratio, 6.9; 95% CI, 2.5– 19.0; *P* < .001) (28).

Boys in our cohort had higher mean MetS risk scores than girls, independent of early onset obesity and obesity status at adolescence. These results are similar to our prior findings (29) and those of US national data, in which adolescent boys were more likely to have MetS risk factors than adolescent girls (9). A recent systematic review of the prevalence of MetS in children and adolescents from 12 countries in North America and South America also found a higher prevalence of MetS among boys (29). Adolescent boys also manifested higher fasting blood glucose, higher blood pressure, and lower HDL cholesterol levels than adolescent girls. These CVD risks were primarily observed in Mexico, Canada, Colombia, and the United States (30).

This research emphasizes the value of studying longitudinal cohorts and the relevance this study’s cohort to obesity in early childhood and adolescent health outcomes among Chileans. In addition to the longitudinal study design, this study has several strengths, such as the uniqueness of a Chilean cohort of infants followed successfully to adolescence, inclusion of a relatively large group of healthy infants, and good participant retention. Another strength is that the evaluation was conducted at a nutrition research center by highly trained study personnel. Furthermore, the use of a sex-specific and race-specific continuous MetS risk score is a study strength. Although continuous scores were previously developed, the methodology followed by Gurka et al and applied in this study, acknowledges correlations between the MetS components, accounting for MetS component correlation differences by sex and race/ethnicity (25).

A limitation of this study is that it may not be generalizable to other populations. The participant sample was restricted to infants who weighed 3 kg or more at birth. Thus, we cannot infer whether these relationships translate to preterm or low-birthweight infants. Also, probably because data on birthweight were restricted, we probably did not observe a relationship between birthweight and obesity. Generalization to higher-income or poverty groups is also restricted. Another limitation is lack of anthropometric data between measurement waves, thus placing participants in a BMI category at time of measurement, which might have been different a year before or after measurement. Additionally, data were unavailable on maternal or paternal obesity status, diet intake, and direct physical activity measures. Although we attempted to minimize unmeasured confounding in our study by including measures on recognized potential confounders, such unmeasured risk factors could confound the relationship between early onset obesity and MetS risk.

Notwithstanding these limitations, our findings add to the literature on early life determinants, in particular determinants related to the long-term effects of early onset obesity on MetS or other CVD-related risk factors. Future research should be conducted in populations with various races and ethnicities to substantiate these findings and address a key public health problem.

Our results underscore the public health implications of early childhood obesity for health outcomes later in life. The findings provide evidence for a clinically meaningful and significant association between early onset obesity and MetS risk score in this Chilean cohort. The results of this study emphasize the importance and need for early detection of childhood obesity and effective public health interventions.
